# Supplementary figures and images for: Impact of a candidate vaccine on the dynamics of salmon lice (Lepeophtheirus salmonis) infestation and immune response in Atlantic salmon (Salmo salar L.)
Source: PLoS One. 2020 Oct 2;15(10):e0239827. doi: 10.1371/journal.pone.0239827 (PMC7531828; doi:10.1371/journal.pone.0239827)

## Skin

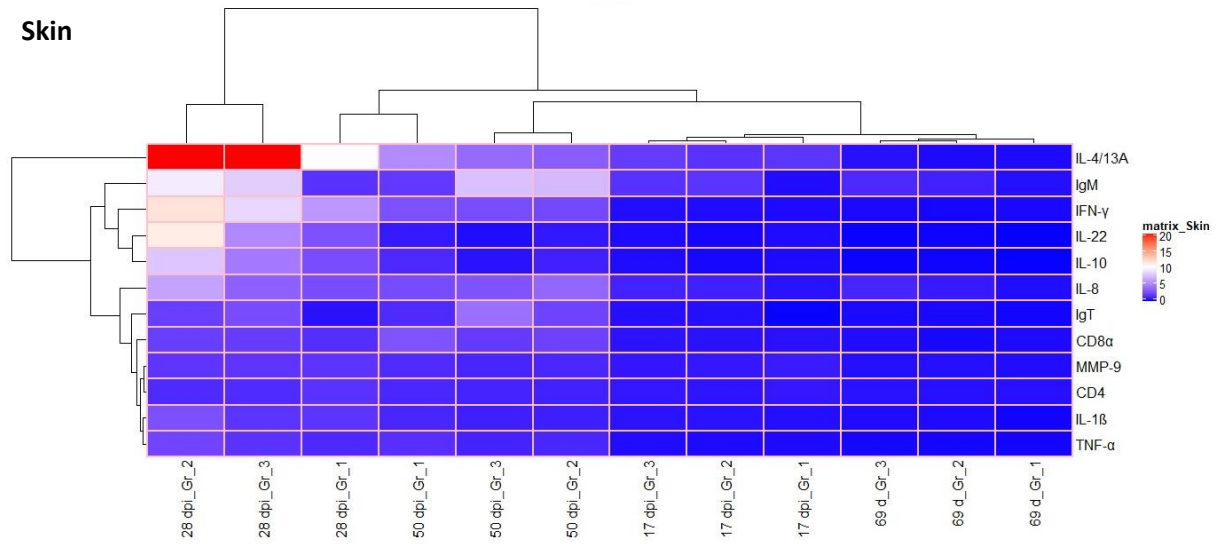

## Head kidney

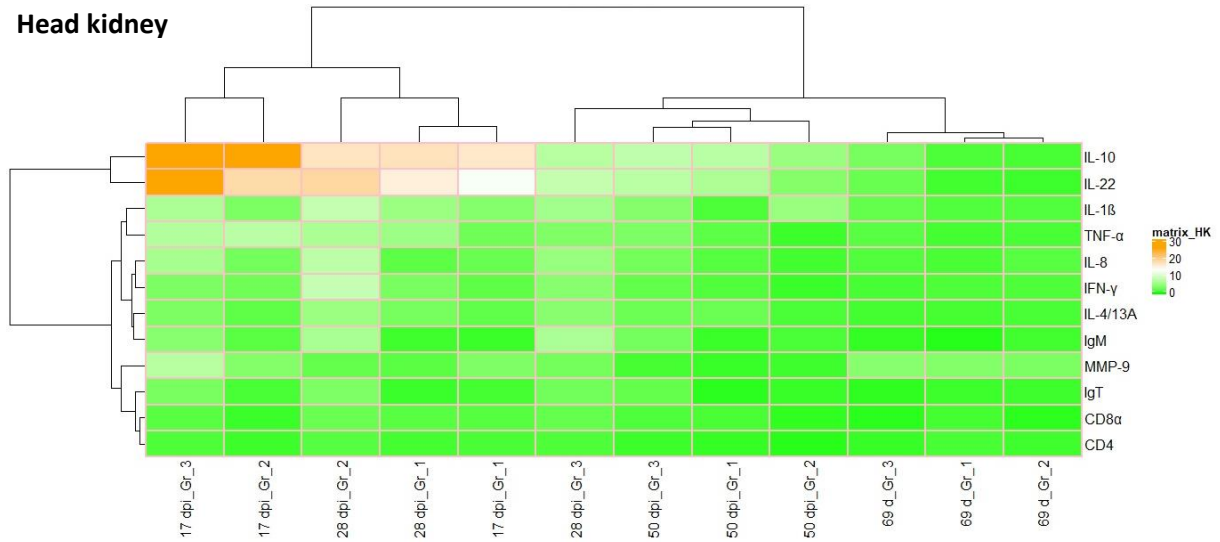

## Spleen

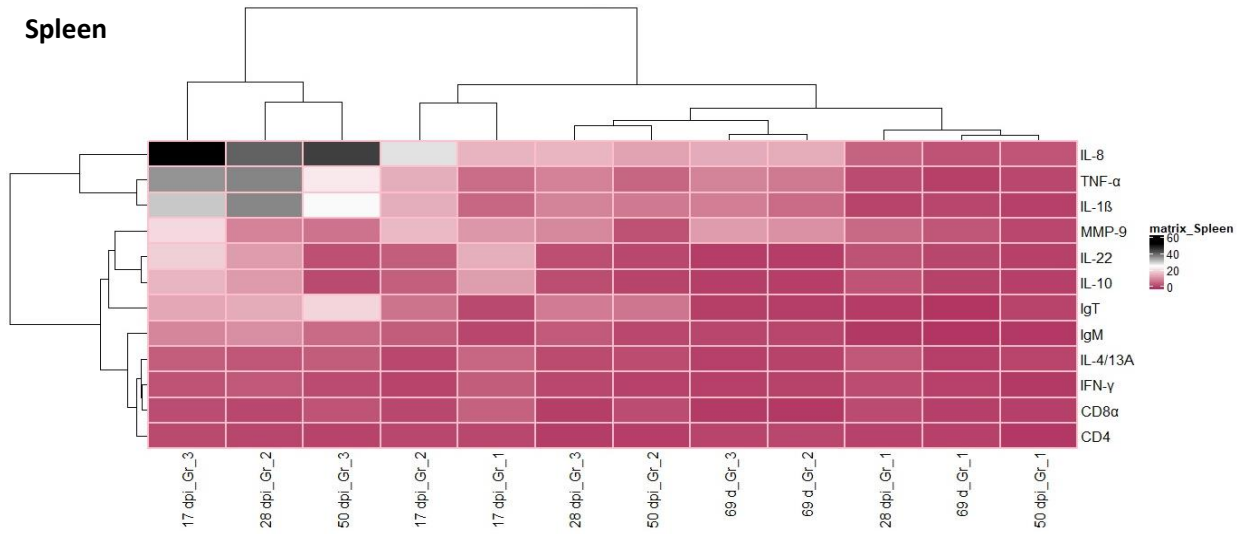

Supplement: S1 Fig — The rows represent gene expression and the column represents different sampling points within respective groups. Group details: Group 1 is control group; Group2 received ip injection of adjuvant emulsified vaccine antigen; Group 3 received ip injection of adjuvant emulsified vaccine antigen + bath immunization. (PDF) [file pone.0239827.s001.pdf]

# PCA analysis- Head kidney

A

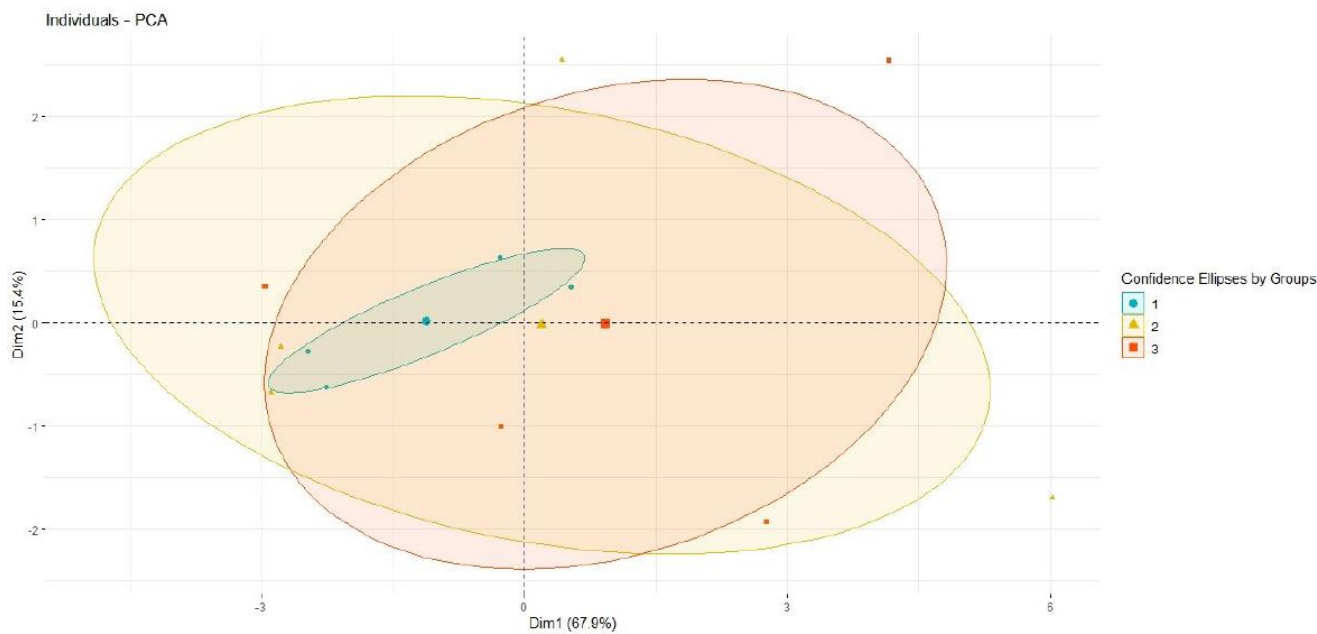

B

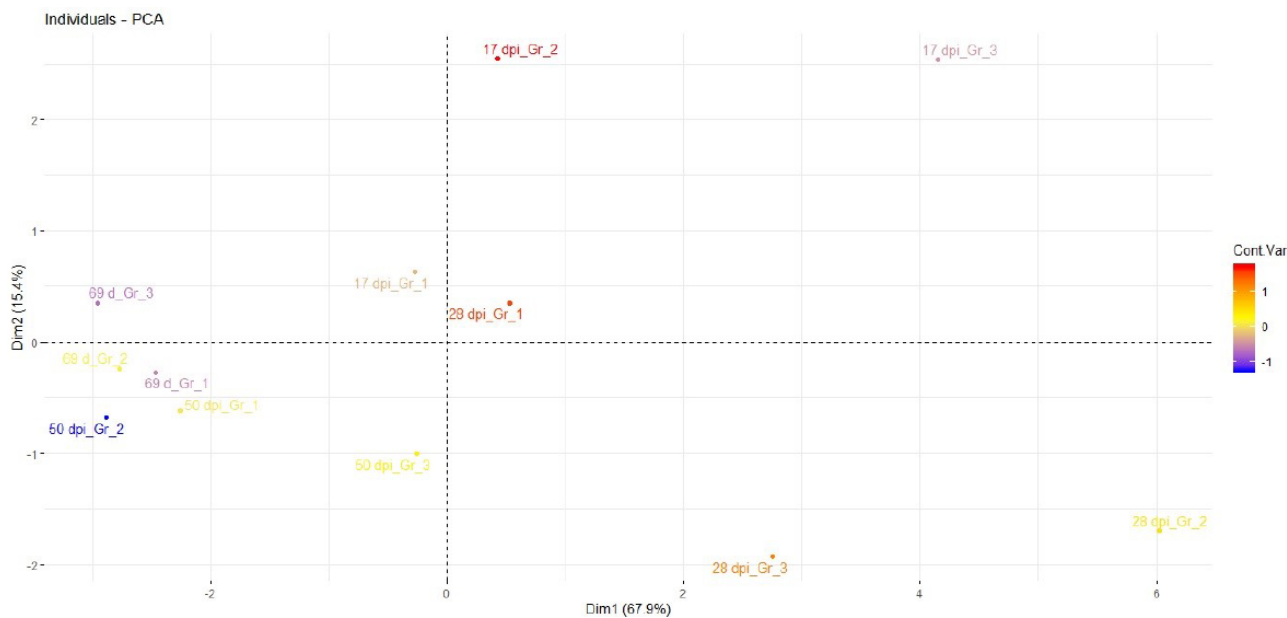

C

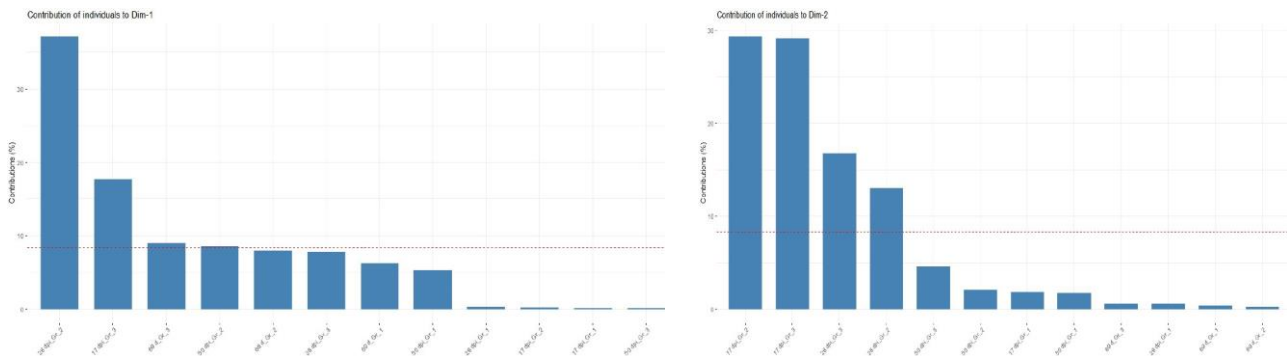

D

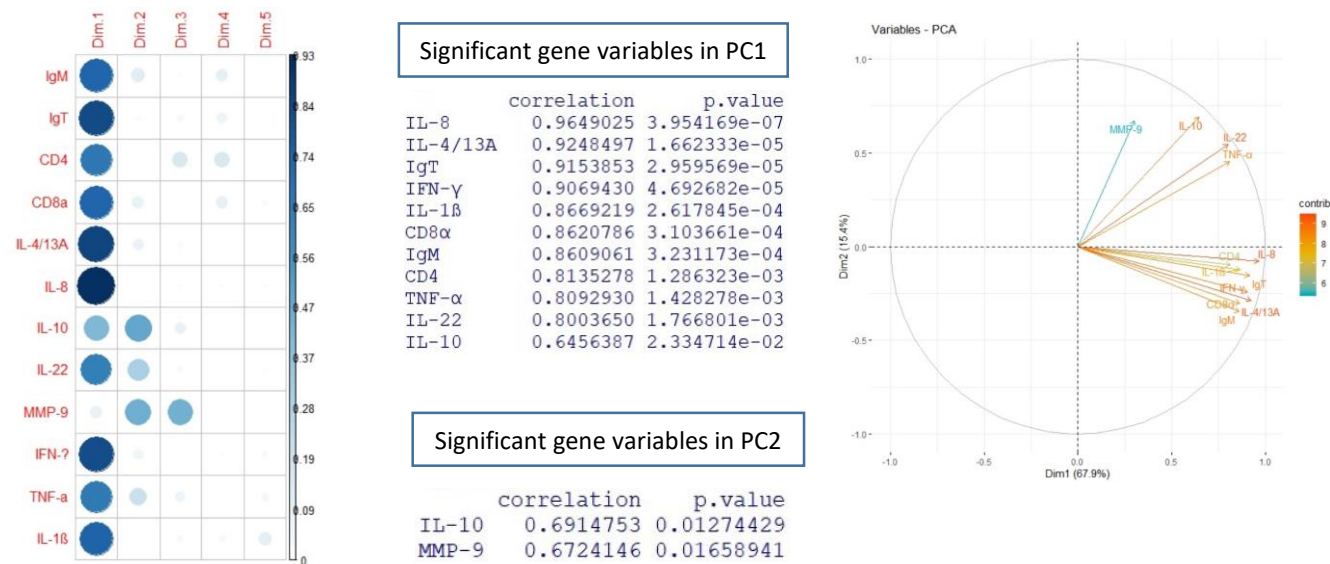

Supplement: S2 Fig — PCA analysis of head kidney samples from vaccinated (group 2 and 3) and only adjuvant vaccinated (group 1) groups at 0, 17, 28 and 50 dpi (A and B). Analysis was based on mean fold-changes of all genes for each individual sample at each sampling point (smaller symbols) relative to the unvaccinated control. The ellipses indicate the group dispersion/variability from the centroid (larger symbols) calculated using all individual fold-changes values/group (A). (C) shows the contribution of sampling points to different components. (D) shows the contribution of genes on different components and the significant genes contributing in principal component 1 and 2. (PDF) [file pone.0239827.s002.pdf]

# PCA analysis- Spleen

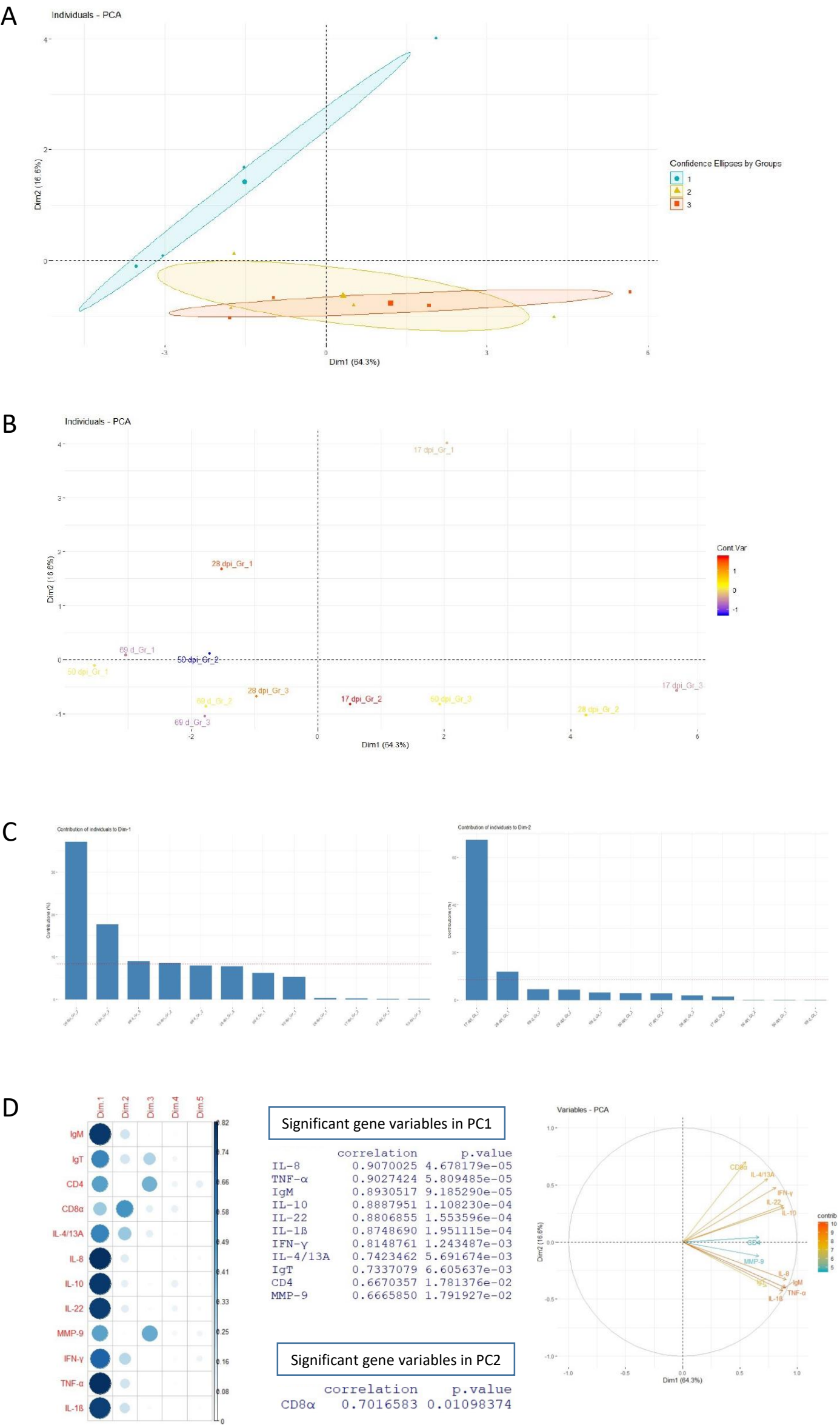

Supplement: S3 Fig — PCA analysis of spleen samples from vaccinated (group 2 and 3) and only adjuvant vaccinated (group 1) groups at 0, 17, 28 and 50 dpi (A and B). Analysis was based on mean fold-changes of all genes for each individual sample at each sampling point (smaller symbols) relative to the unvaccinated control. The ellipses indicate the group dispersion/variability from the centroid (larger symbols) calculated using all individual fold-changes values/group (A). (C) shows the contribution of sampling points to different components. (D) shows the contribution of genes on different components and the significant genes contributing in principal component 1 and 2. (PDF) [file pone.0239827.s003.pdf]

A

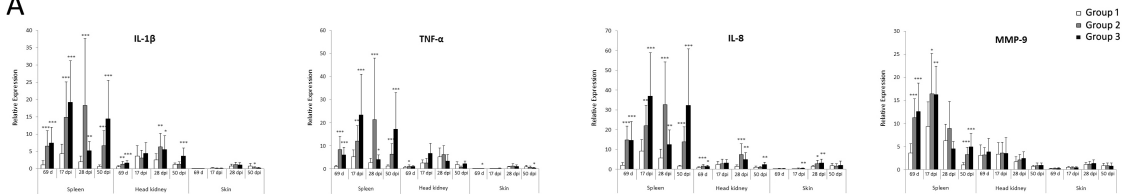

B

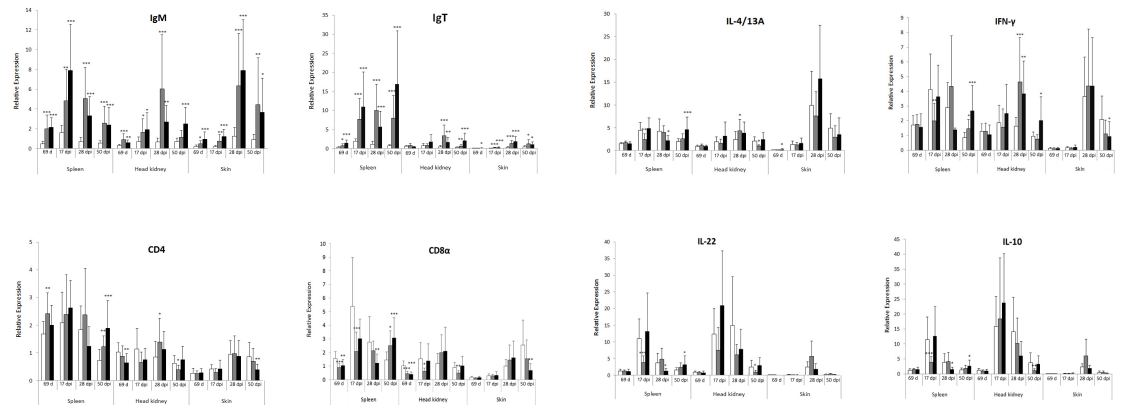

Supplement: S4 Fig — Transcript levels of the pro-inflammatory cytokines (A) and immune genes (B) in spleen, head kidney and skin at different sampling points: 69 days from first vaccination (69d) or zero day challenge and after challenge (dpi: days post infestation), were analysed by real-time QPCR. The QPCR data were normalized to the geometric mean of the 3 house-keeping genes (EF-1a, 18S and β-actin) and expression is relative to the pre-immunized level. Fold change was calculated using the primer efficiency. Data shown represent the mean ± SD of experiments performed in triplicate, n = 18 fish/group (6 fish/replicate). Statistical analysis was carried out using one-way ANOVA or Kruskal Wallis test followed by Tukey or Dunn's Multiple Comparison compared to control group (*P < 0.05, **P < 0.01, ***P < 0.001). Group details: Group 1 is control group; Group2 received ip injection of the adjuvant emulsified vaccine antigen; Group 3 received ip injection of adjuvant emulsified vaccine antigen + bath immunization. (PDF) [file pone.0239827.s004.pdf]

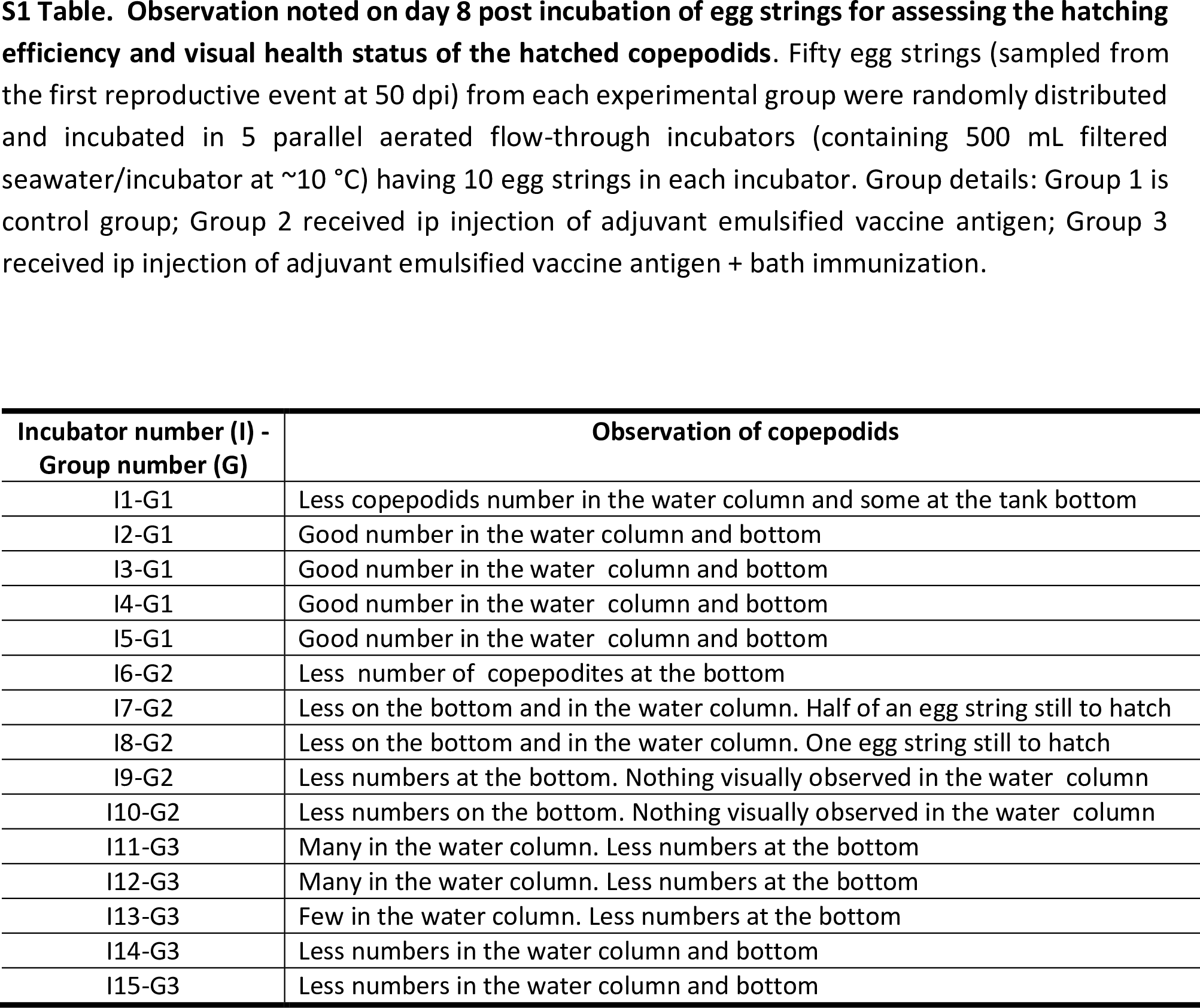

Supplement: S1 Table — Fifty egg strings (sampled from the first reproductive event at 50 dpi) from each experimental group were randomly distributed and incubated in 5 parallel aerated flow-through incubators (containing 500 mL filtered seawater/incubator at ~10°C) having 10 egg strings in each incubator. Group details: Group 1 is control group; Group 2 received ip injection of adjuvant emulsified vaccine antigen; Group 3 received ip injection of adjuvant emulsified vaccine antigen + bath immunization. (TIF) [file pone.0239827.s005.tif]
