# Supplementary material for: Impact of a candidate vaccine on the dynamics of salmon lice (Lepeophtheirus salmonis) infestation and immune response in Atlantic salmon (Salmo salar L.)
Source: PLoS One. 2020 Oct 2;15(10):e0239827. doi: 10.1371/journal.pone.0239827 (PMC7531828; doi:10.1371/journal.pone.0239827)
Supplement: Attachment — Submitted filename: Response to Reviewers.docx [file pone.0239827.s008.docx]

**Response to Reviewer**

Reviewer #2: The authors have generally done a good job of revising the manuscript, and I have only two substantive concerns remaining (one of which still affects the overall vaccine efficacy estimate). I’m confident that both concerns can be addressed to my satisfaction.

The writing could still benefit from some copy editing, but I’ll leave this for the authors and/or journal to manage.

Well done to the authors for the effort they’ve put into this trial.

**Authors response:** Thanks a lot. We really appreciate reviewer’s effort for good self-explanatory advices and comments.

1) Regarding lice and egg string loss during netting

Original comment:

L162: It is good that counting was done this way, but how were fish sampled? My experience is that most lice are actually lost during netting and anaesthesia rather than during counting. This paragraph should say how the sampling took place. For example, were the tanks sedated before sampling (this reduces the number of lice lost during netting), were fish netted or removed some other way (again, affects how many lice are lost), were the 10 fish per tank anaesthetised together in a bucket or separately, was the anaesthetic water checked for detached lice, etc.). This shouldn’t qualitatively change the vaccine effect, but it may change our understanding of infestation density across all three groups (especially because lice levels decreased markedly over time).

Authors’ response:

As per reviewer suggestion, the paragraph was modified accordingly in L170-178 as mentioned below: “Fish were taken out one at a time gently by handle fishing net from the stocking tanks and transferred in a bucket containing an overdose of anaesthetic water (0.01% benzocaine). Care was taken for the minimum loss of lice through netting. The net was simultaneously checked for the detached lice. One fish per bucket was anaesthetized before lice counting. To avoid counting error of detached lice due to anaesthesia and handling, counting of chalimus at 17 dpi, pre-adults at 28 dpi and adults at 50 dpi on individual parasitized fish were performed under water in a white tray. After lice counting from each fish/tray, the remaining water in the respective tray and bucket were checked for detached lice.” Moreover, we have experienced that after sedation of fish, lice is detached very quickly from the fish. Therefore, fish were taken out first one by one through handle net and then anaesthetised in a white bucket and simultaneously the net was checked for any detached lice on the net in anaesthetic water.

New comment:

This is a better description of the sampling protocol, although I’m still not convinced that the authors are able to prevent loss of any lice or egg strings during netting. As I noted in my original comment, I realise that this does not matter much for the measured vaccine efficacy because all groups received the same handling, but in a study that directly concerns infestation density and infestation persistence, likely sources of lice loss during sampling should be acknowledged. It is well known that netting salmon (and especially unsedated salmon) causes some adult lice and egg strings to be dislodged from abrasion by the mesh, and netting “gently” or “with care” doesn’t change this, especially if the fish is struggling. I would therefore like the authors to include a note either acknowledging this or explaining how they can be sure that none were lost. E.g. (i) maybe the net mesh was fine enough to capture lice and egg strings that might have been dislodged, (ii) maybe they are confident that they would see any dislodged egg strings in the tank after netting (detached lice would likely reattach to another host in the tank – something I’ve seen before), or (iii) acknowledge that some lice and egg strings could have been lost before the fish was transferred to the anaesthetic bucket.

A small point on wording: Change “handle fishing net” to your choice of “hand net”, “dip net” or “scoop net”

**Authors Response:** As suggested by the reviewer, we have acknowledged lice loss during sampling and added text accordingly in L171-174 of the revised manuscript in.

“**All fish were treated the same and were handled gently. The hand nets were checked for detached lice and the net mesh was fine enough to capture lice if any lice would have fell off. However, we cannot exclude the possibility that some egg strings or lice would have been detached from the fish or lost in the tank during handling (netting).**”

As advised, we changed “handle fishing net” to “hand net”

2) Regarding the vaccine efficacy formula

Original comment:

L186: This formula seems problematic to me: “Vaccine efficacy (%) = 100 x [1-(NCh x NPA x NF x NM x FE x NE x LE x CC)]”. Unless I misunderstand, it looks like repeated counting of the same effect. For example, there is no need to include the immature stages here – losses of lice at all stages will accumulate and be ultimately represented by the difference in the density of adult females. You can then multiply that effect by effects on reproductive output per female. The study cited (Rodríguez-Mallon et al. 2015) used a similar formula but using different parameters, so does not support the usage here. I also disagree with the use of egg string number (NE), as this is more likely to be a sampling artefact than a biological effect (see also L256). Moreover, wouldn’t F1 copepodid counts also incorporate differences in egg string length (LE) if that variable is important? Unless the authors can explain why this isn’t repeated counting of the same effects, I recommend using a much simpler and more defensible formula to calculate an overall effect, representing how many female lice survive to maturity and how many larvae they produce: Vaccine efficacy (%) = 100 x [1-(FE x CC)]. Note, the CC parameter should be corrected for the number of egg strings collected per female (copepod count / number of egg strings).

Authors’ response:

Taking into account the reviewer suggestion, we assumed the proposed formula of vaccine efficacy but include the parameter LE as shown below:

Vaccine efficacy (%) = 100 x [1-(FE x LE x CC)].

LE: length of egg strings in vaccinated group/length of egg strings in control group. The length of egg strings are considered representative of how many eggs were produced, because it is almost impossible to count the eggs one by one.

CC: F1 generation copepodids count from vaccinated group/F1 generation copepodids count in control group. It is an indicator of how many larvae are produced from a fixed amount of egg strings. We think that the correction of CC is not valid since we placed the same number of egg strings per group (50 egg strings per group divided in 5 incubation chambers with 10 egg string in each one) in the incubation chambers for hatching.

With the new formula, vaccine efficacy was 58% for group 2 and 20% for group 3. This was corrected in the text.

New comment:

I agree with the authors that CC should not be corrected for the number of egg strings (it is now clear that the same number of egg strings were used per group).

However, the inclusion of LE still seems incorrect. I agree that LE is a fair proxy for number of eggs, and is an interesting variable to measure and report in the paper, but I don’t agree with it being used in this way in the formula. This is because the CC parameter already accounts for differences in the number of eggs produced – all else being equal, fewer eggs per egg string will result in fewer F1 larvae per egg string (this contributes to the observed effect on CC). Therefore, the vaccine effect on LE is already contained within the effect on CC, and multiplying the two parameters within the same formula is double-counting the FE effect and inflating the overall effect by a few %.

So I have to push for my original recommendation for the formula:

Vaccine efficacy (%) = 100 x [1-(FE x CC)].

Alternatively, the authors could include FE and CC, but in a different form where LE is the effect on number of eggs produced, and CC/LE is the effect on copepodid production from a given number of eggs (not egg strings). This avoids double-counting and gives the same result as 100 x [1-(FE x CC)], but explicitly shows the importance of LE by partitioning the vaccine effects on reproduction into (i) number of eggs per egg string, and (ii) number of larvae per egg:

Vaccine efficacy (%) = 100 x [1-(FE x LE x (CC/LE))]

For simplicity, CC/LE could be assigned to a new parameter called, for example, CE (copepodids per egg) to simplify the formula. The formula would then be:

Vaccine efficacy (%) = 100 x [1-(FE x LE x CE)].

Where FE is the effect on female survival to maturity, LE is the effect on fecundity of adult females (using egg string length as a proxy for fecundity), and CE is the effect on hatching and survival of F1 offspring to the copepodid stage.

**Authors Response:** First, we appreciate reviewer’s effort for good self-explanatory advices and comments.

We agree with reviewer’s original recommended formula as well as the alternative formula “Vaccine efficacy (%) = 100 x [1-(FE x LE x CE)]”. Both the original and the alternative formula as suggested by the reviewer gave exactly the same value of vaccine efficacy. i.e 56% for group 2 and 25% for group 3.

Here, we preferred to use the alternative formula as suggested by the reviewer, which accounts the effect on female fecundity as well as on hatching and survival of F1 offspring to the copepodid stage. The corrected vaccine efficacy formula and values are included in the revised manuscript.

(Just to mention, there was a minor error in calculation for Gr 3 in the efficacy and in Table 2. This has been corrected in the revised version)
